# Influence of antiviral treatment in hepatitis C patients on metabolism and fibrosis process

**DOI:** 10.3389/fmed.2026.1729835

**Published:** 2026-02-25

**Authors:** Malwina Jędrysik, Krzysztof Tomasiewicz, Beata Chełstowska, Magdalena Tudrujek-Zdunek, Filip M. Szymański

**Affiliations:** 1 Department of Biochemistry and Laboratory Diagnostics, Faculty of Medicine, Collegium Medicum, Cardinal Stefan Wyszynski University in Warsaw, Warsaw, Poland; 2 Department of Infectious Diseases, Medical University of Lublin, Lublin, Poland; 3 Department of Civilization Diseases, Faculty of Medicine, Collegium Medicum, Cardinal Stefan Wyszynski University, Warsaw, Poland

**Keywords:** DDA, drug, fibrosis, HCV, metabolism

## Abstract

**Background:**

Chronic Hepatitis C Virus (HCV) infection is associated with systemic metabolic disturbances, including glucose intolerance, lipid dysregulation, and inflammation, accelerating liver fibrosis and increasing hepatocellular carcinoma risk. Biomarkers such as Glucagon-Like Peptide-1 (GLP-1), Fatty Acid-Binding Protein 1 (FABP-1), Monocyte Chemoattractant Protein-1 (MCP-1), Angiopoietin-Like Protein 6 (ANGPTL6), ibroblast Growth Factor 19 (FGF-19), and ghrelin offer insights into these mechanisms and may reflect the impact of antiviral treatments.

**Objectives:**

This study evaluated the effects of two direct-acting antiviral (DAA) regimens—Glecaprevir/Pibrentasvir (G/P) and Sofosbuvir/Velpatasvir (S/V)—on metabolic and inflammatory biomarkers in 70 HCV-infected patients with comorbidities including obesity, type 2 diabetes, and hypertension.

**Methods:**

Serum biomarker levels were assessed pre- and post-treatment using the Luminex FlexMAP 3D system. Patients were stratified by treatment type, fibrosis stage (F0–F4), and baseline cholesterol. Liver stiffness was evaluated via FibroScan.

**Results:**

Both regimens induced significant biomarker changes. ANGPTL6, FGF-19, ghrelin, total cholesterol, HDL, and non-HDL increased, while FABP-1 and MCP-1 decreased, indicating reduced inflammation and lipid stress. Stronger effects were seen in patients with lower baseline cholesterol and with S/V in advanced fibrosis. G/P showed marked anti-inflammatory effects in early fibrosis.

**Conclusion:**

DAA therapy significantly alters metabolic and inflammatory biomarkers in HCV patients, with regimen- and fibrosis stage-specific effects. S/V may provide broader metabolic benefits in advanced disease, while G/P offers stronger anti-inflammatory responses earlier, supporting personalized treatment approaches.

## Graphical abstract

**Figure:**
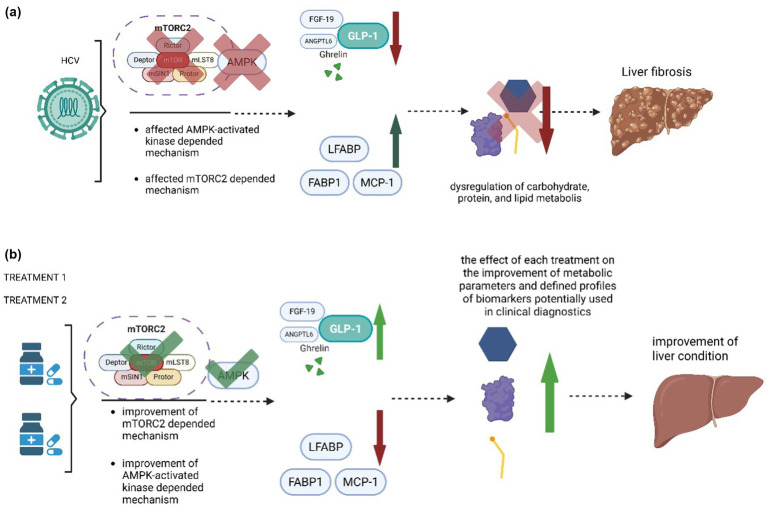
(a) The figure shows the effect of HCV on disrupting mTORC2 and AMPK-dependent cellular mechanisms, resulting in deregulated carbohydrate, protein and lipid metabolism and liver fibrosis. (b) The figure shows the effect of antiviral treatment on improving carbohydrate, protein, and lipid metabolism, and consequently to improving liver health. Created with BioRender.com.

## Introduction

Hepatitis C virus (HCV) infection is a systemic disease associated with diverse extrahepatic disorders including atherosclerosis, glucose and lipid metabolic disturbances, alterations in the iron metabolic pathways (1). The inseparable associations between chronic HCV infection and metabolic disease listed above are specifically important considering the increasing prevalence of obesity and metabolic syndrome in the society (2). This study aimed to elucidate the molecular mechanisms underlying the recurrent clinical observations seen in patients undergoing antiviral therapy. HCV infection inhibits AMPK activity and activates the mTORC2 pathway, promoting viral replication and disrupting hepatocyte metabolism; following successful antiviral therapy (DAAs), partial restoration of AMPK function and normalization of mTORC2 activity are observed, although full recovery of these pathways depends on the extent of liver damage (3, 4). The AMP-activated protein kinase (AMPK) and mechanistic target of rapamycin (mTOR) pathways form a key regulatory axis controlling cellular energy balance and metabolic adaptation. In chronic hepatitis C virus (HCV) infection, viral proteins disrupt these pathways, leading to suppression of AMPK activity and persistent activation of mTOR signaling, which promotes hepatic lipid accumulation, insulin resistance, and chronic inflammation. Dysregulation of the AMPK/mTOR axis has also been linked to activation of hepatic stellate cells and progression of liver fibrosis, connecting metabolic stress with HCV-related liver disease (5, 6). Understanding the cellular and molecular interplay is crucial, as combined metabolic and infectious disturbances accelerate liver fibrosis and liver cancer development (5). Given the potential mechanistic pathways, we chose previously characterized biomarkers to evaluate alterations in their serum levels and their responsiveness to antiviral therapy.

Glucagon-like peptide-1 (GLP-1) is a gut-derived incretin hormone that stimulates insulin secretion. It is released postprandially from intestinal L-cells and neurons in the caudal nucleus of the solitary tract, reaching the hypothalamus (7). In the pancreas, GLP-1 receptor activation enhances insulin secretion, increases insulin sensitivity, and inhibits glucagon release. This improves insulin signaling in the liver, muscle, and adipose tissue, enhancing glucose uptake, reducing hepatic gluconeogenesis, and improving steatosis. GLP-1 also modulates immune responses, reducing systemic inflammation and steatohepatitis.

In HCV-infected patients, serum GLP-1 levels are significantly reduced compared to controls, suggesting a role in HCV-related glucose intolerance (8). HCV not only affects liver function but also disrupts glucose metabolism and promotes insulin resistance (9). Altered GLP-1 levels and signaling in HCV patients may worsen metabolic disturbances associated with chronic liver disease (10, 11). Moreover, GLP-1 receptor agonists like liraglutide have been shown to inhibit HCV replication via AMPK- and TORC2-dependent pathways (12, 13), highlighting their therapeutic potential in HCV-related metabolic dysfunction.

Liver fatty acid-binding protein (FABP-1), abundant in hepatocytes, is a sensitive marker of liver injury. It participates in fatty acid transport and, due to its small size, appears in circulation earlier than larger proteins like AST (47.5 kD) and ALT (96 kD), even with minimal cell damage (14). Changes in L-FABP levels in chronic hepatitis C infection may indicate acute liver damage (FABP1 has a short plasma half-life of 11 min before renal clearance), according to preliminary research, albeit its diagnostic utility in this regard is yet unclear (15, 16). Therefore, understanding the intricate molecular processes and clinical implications of the interplay between GLP-1 signaling, HCV infection, and L-FABP holds great promise for advancing our understanding of viral pathogenesis and metabolic dysfunction (17, 18).

Another marker that is involved in the energy metabolism of cells, including hepatocytes, and which we considered in the context of HCV infection is ghrelin. This hormone is involved in energy metabolism, food intake, and glucose homeostasis and many studies have assessed whether ghrelin acts as an independent signal of adiposity or as a downstream mediator of leptin, affecting energy balance (19). Although not extensively studied, elevated ghrelin levels in cirrhosis and hepatocellular carcinoma suggest a role in anorexia-cachexia. Ghrelin may also protect against steatosis, inflammation, oxidative and ER stress, and support autophagy and immune responses, improving liver disease progression (20, 21).

Considering the mechanisms of the inflammatory response in the course of HCV infection, we took into account the concentration of the monocyte chemo-attractant protein-1 (MCP-1) which is a member of a large family of chemokines known to be important soluble mediators of innate immunity and tissue inflammation. MCP-1 has a key role in controlling monocyte/macrophage migration (22). MCP-1 help in the recruitment of activated macrophage into the liver tissue in case of hepatotoxicity and hepatic inflammation and in HCV-infected patients with severe fibrosis, the mRNA MCP-1 liver expression was higher than in mild liver fibrosis patients. HCV infection is associated with increased hepatic expression of MCP-1 (23, 24).

It is also worth to mention about additional marker, Angiopoietin-like protein 6 (ANGPTL6), which is a member of the angiopoietin-like protein family, primarily synthesized in the liver. It exhibits pleiotropic effects involving both metabolic and vascular functions (25). ANGPTL6 regulates glucose and lipid metabolism by enhancing insulin sensitivity and energy expenditure. Its deficiency causes insulin resistance, dyslipidaemia, and obesity (25, 26). Additionally, ANGPTL6 supports angiogenesis and tissue repair processes, making it a potentially important factor in liver remodelling after inflammatory damage.

The last worth to mention about marker is known as Fibroblast Growth Factor 19 (FGF-19), which is a hormone produced in the small intestine (in the ileum) in response to bile acid presence (27). It acts via the FGFR4 receptor in the liver, playing a key role in regulating bile acid, glucose, and lipid metabolism. FGF-19 suppresses bile acid synthesis in the liver by downregulating the CYP7A1 gene, promotes glycogen storage, and lowers blood glucose levels. Dysregulation of FGF-19 secretion has been observed in various metabolic conditions, such as type 2 diabetes, obesity, and liver diseases, indicating its potential role as a biomarker for metabolic dysfunction in the gut-liver axis (26–28).

All these biomarkers were collated and analysed in relation to recognized biochemical parameters of metabolic disorders: glucose levels, lipid fractions, and triacylglycerol levels, and in relation to parameters of inflammation, markers of myocardial performance and comorbidities.

Sofosbuvir/Velpatasvir (S/V) and Glecaprevir/Pibrentasvir (G/P) are direct-acting antivirals (DAAs) targeting key steps in the HCV lifecycle (29). S/V combines sofosbuvir, an NS5B polymerase inhibitor, and velpatasvir, an NS5A inhibitor, effectively treating genotypes 1–6 and achieving high SVR rates, even in cases like compensated cirrhosis (30). G/P includes glecaprevir, an NS3/4A protease inhibitor, and pibrentasvir, an NS5A inhibitor, also active against genotypes 1–6 and suitable for patients with severe renal impairment without dose adjustment (31, 32).

Both regimens require monitoring for drug interactions and potential HBV reactivation. Their efficacy across diverse patient groups underscores their therapeutic value. NS3/4A protease inhibitors are also used in HIV therapy; however, metabolic effects differ. In HIV, protease inhibitors can cause lipodystrophy due to immune activation and mitochondrial toxicity, especially with NRTIs (33). In contrast, HCV therapy does not cause similar metabolic disturbances, as HCV primarily affects the liver. Thus, this paper aims to comprehensively explore these multifaceted relationships, shedding light on novel therapeutic avenues and insights into the management of chronic liver disease and associated metabolic comorbidities.

The aim of this study was to evaluate the impact of direct-acting antiviral therapy on metabolic and inflammatory biomarkers in patients with chronic HCV infection and to investigate how viral eradication affects pathways involved in lipid, carbohydrate, and protein metabolism as well as liver fibrosis. In addition, we assessed whether the observed changes differ according to the antiviral regimen used, the stage of liver disease, and baseline lipid profile.

## Patients

The study included patients from the Infectious Diseases and Hepatology Outpatient Clinics at the University Clinical Hospital in Lublin. Clinical evaluations were conducted before starting interferon-free direct-acting antiviral (DAA) therapy and 12 weeks after treatment (SVR12), the standard point for assessing sustained virological response. Treatment followed the National Health Fund (NFZ) Drug Program and Polish HCV Expert Group guidelines. Two regimens were used: 12 weeks of S/V in 35 patients and 8 weeks of G/P in another 35. Eligibility required detectable HCV RNA via quantitative NAT, along with routine labs (CBC, ALT, bilirubin, HBsAg, anti-HBc, anti-HIV). Liver fibrosis was assessed by transient elastography (FibroScan), reported in kPa and staged using the METAVIR system (F0–F4). Patients with liver disease of other etiologies, including HBV infection, autoimmune liver diseases, and significant alcohol consumption, as well as those after liver transplantation, were excluded from the study.

The multiplex biomarkers analysis of serum taken from 70 patients with HCV were performed using Luminex FlexMap 3D technique. In each serum sample concentration of six biomarkers (ANGPTL6, FGF-19, ghrelin, MCP-1, FABP-1, GLP-1) were measured.

### Experimental design, materials and methods

Serum samples collected from 70 consecutive HCV-positive patients clinically diagnosed with additional comorbidities such as hypertension, type II diabetes and obesity. Prior informed consent was obtained from each patient.

The data shows the biomarker profiling results acquired in a cohort of 70 HCV patients clinically diagnosed with additional comorbidities using the FlexMAP 3D (Luminex) platform using the Milliplex Human Metabolic Hormone Panel V3 and Milliplex Map Human Liver Protein Magnetic Bead Panel-Metabolism Multiplex Assay. Measurements of the analysed parameters (ANGPTL6, FGF-19, ghrelin, MCP-1, FABP-1, GLP-1) using the FlexMAP 3D (Luminex) platform were prepared and performed in duplicates. Biomarkers levels against the different time points which indicate the response to the given treatment and against the HCV viral load were analysed. Two different treatments were given to this population. Half of them (*n* = 35) received S/V treatment and the second half received G/P treatment (*n* = 35). All analysed parameters were assessed across patient groups stratified by liver stiffness measurements obtained via FibroScan ® examination. FibroScan® is a non-invasive, clinically validated medical device that measures liver stiffness (as an indicator of fibrosis) and the controlled attenuation parameter (CAP®, reflecting liver steatosis). The procedure is safe, rapid, and requires no tissue biopsy. Measurements were interpreted by trained medical specialists in accordance with established clinical guidelines.

## Results

Changes in parameter levels before and after treatment were analysed using the Wilcoxon signed-rank test for paired variables, chosen due to non-normal data distribution and small subgroup sizes. Analyses were performed across the whole cohort by fibrosis stage (F0–F4), separately for each stage (F1–F4) regardless of treatment, and by fibrosis stage with treatment type (e.g., F1 G/P, F1 S/V). The F0 group was included only in the overall analysis due to its small size. This approach enabled a detailed evaluation of treatment effects by disease severity and regimen.

1) Concentration of studied parameters in whole studied population of patients and in studied groups, regardless of treatment and fibrosis stage:

ANGPTL6, FGF-19, Ghrelin, GLP-1, MCP-1, and FABP-1 parameters were measured in the entire group of patients (34–38). The differences in the medians of all parameters in the patients before and after treatment regardless of used treatment are shown in Table A1 (see Appendix 1).

Following antiviral treatment, a statistically significant increase in the median concentration of ANGPTL6 was observed, rising from 315.80 pg./mL (IQR: 336.3) to 453.30 pg./mL (IQR: 389.60), *p* < 0.0001. Similarly, FGF-19 levels increased from 0.09 (IQR: 0.10) to 0.13 pg./mL (IQR: 0.08), *p* = 0.0344. Ghrelin levels significantly increased from a median of 5.070 (IQR: 7.94) to 7.38 pg./mL (IQR: 24.29), *p* = 0.0264. GLP-1 showed a non-significant reduction from 1.66 (IQR: 2.52) to 1.39 pg./mL (IQR: 1.81), *p* = 0.1926. MCP-1 decreased non-significantly from 133.10 (IQR: 83.78) to 125.10 pg./mL (IQR: 57.68), *p* = 0.0782. Finally, FABP-1 levels also showed a statistically significant reduction, from 0.08 (IQR: 0.15) to 0.04 pg./mL (IQR: 10.22), *p* = 0.042.

Additionally, in studied group we also analysed comorbidities like diabetes, hypertension, obesity, concentration of lipids (total cholesterol, triglycerides), parameters of inflammation (CRP, WBC).

In the S/V-treated group, 3 patients had diabetes and 9 had hypertension and 6 had obesity (BMI > 30). In the G/P-treated group (35 patients), apart from obesity observed in 6 patients, no other comorbid conditions were present. Key biochemical parameters reflecting metabolic status—total cholesterol, triglycerides, HDL, non-HDL, CRP, and BNP—were analysed. Statistical results before and after treatment are shown in Table A1. Following antiviral therapy, a statistically significant increase in median total cholesterol was observed (147.6–177.5 mg/dL, *p* < 0.0001), accompanied by significant elevations in HDL (35.55–43.3 mg/dL, *p* < 0.0001) and non-HDL cholesterol (115.8–129.4 mg/dL, *p* < 0.0001). No significant changes were detected in CRP or BNP levels. Prior to treatment, 4.2% of patients exhibited lower cholesterol, whereas 34.2% demonstrated increased levels post-treatment. HDL concentration increased in 91% of patients, and triglycerides were elevated in 50%. The rise in non-HDL cholesterol was significant irrespective of treatment modality, indicating both regimens contributed to this effect (see Table A2, Appendix 1).

**Figure 1:**
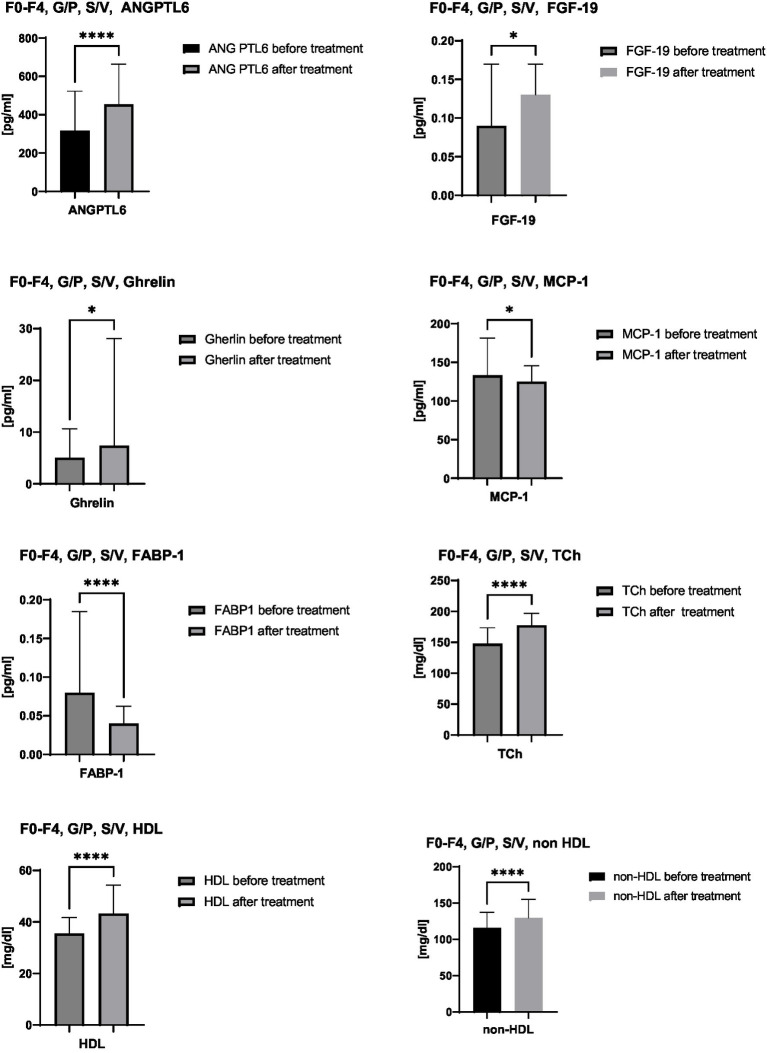
Comparison of serum concentrations of selected parameters in the entire patient population (including fibrosis stages F0, F1, F2, F3, and F4), treated with both G/P (Glecaprevir/Pibrentasvir) and S/V (Sofosbuvir/Velpatasvir). Sample sizes: ANGPTL-6—*N* = 70, FGF-19—*n* = 66; ghrelin—*n* = 69; MCP-1—*n* = 70; FABP-1—*n* = 70; total cholesterol—*n* = 70; HDL—*n* = 70, non-HDL—*n* = 70.

Statistically significant parameters identified in Tables A1 and A2 (see Appendix 1) are depicted in Figure 1. The label “F0–F4” represents the entire cohort across all fibrosis stages. Treatment groups are indicated as “S/V” (Sofosbuvir/Velpatasvir) and “G/P” (Glecaprevir/Pibrentasvir), independent of fibrosis stage. Each panel is annotated with the analysed parameter, illustrating significant changes relative to treatment and fibrosis severity.

**Figure 2:**
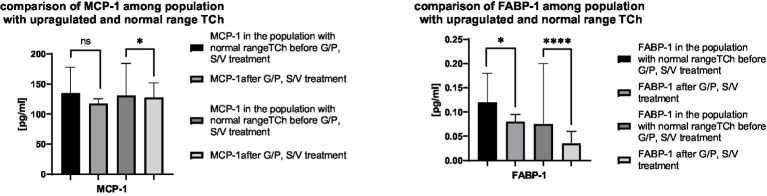
Comparison of serum concentrations of MCP-1 and FABP-1 in the entire patient population (fibrosis stages F0–F4), stratified by baseline cholesterol status. The analysis includes two subgroups: patients with elevated cholesterol levels (>190 mg/dL; *n* = 6) and those with cholesterol within the normal range (*n* = 64).

To further examine the link between lipid metabolism and selected biomarkers, patients were stratified by baseline total cholesterol into two subgroups: elevated (>190 mg/dL) and normal. Biomarkers previously found significant—ANGPTL-6, FGF-19, ghrelin, MCP-1, and FABP-1—were re-analysed within each subgroup. Significant differences were observed for MCP-1 only in the normal cholesterol group, while FABP-1 showed significant changes in both groups, with a stronger effect in the normal range subgroup. These results are shown in Figure 2.

2) Concentrations of the studied parameters in the overall patient population, compared between treatment groups (G/P vs. S/V), irrespective of liver fibrosis stage.

Table 1 shows comparison of statistically significant parameters in at least 1 group, before and after treatment in two group of patients: G/P and S/V group, regardless of the fibrosis stage. The table presents concentrations of parameters including ANGPTL6, FGF-19, ghrelin, MCP-1, FABP-1, TCh, HDL, and Non-HDL [mg/dl], showing median before treatment, IQR before treatment, median after treatment, IQR after treatment, along with *p*-values and statistical significance for each parameter in the G/P and S/V treatment groups.

**Table 1:** Changes in parameters concentration of separated G/P-treated and Sofosbuvir/Velpatasvir-treated group of patients, regardless of the fibrosis stage.

Parameter		G/P						S/V					
		Median before treatment	IQR before treatment	Media`n after treatment	IQR after treatment	*p*-value	Statistical significance	Median before treatment	IQR before treatment	Median after treatment	IQR after treatment	*p*-value	Statistical significance
[pg/ml]	ANGPTL6	218.10	163.10	353.40	235.00	<0.0001	****	495.20	425.60	550.80	432.10	0.0328	*
	FGF-19	0.08	0.08	0.12	0.08	0.0069	**	0.13	0.16	0.14	0.11	0.5131	ns
	ghrelin	4.65	7.55	9.49	48	0.0438	*	5.83	7.965	6.89	8.5	0.2873	ns
	MCP-1	150.20	84.70	134.60	35.40	0.0356	*	117.6	59.76	98.85	63.47	0.1736	ns
	FABP-1	0.09	0.12	0.04	0.03	<0.0001	****	0.05	0.24	0.03	0.06	0.0010	***
[mg/dl]	TCh	149.70	32.90	178.60	41.50	<0.0001	****	146.60	53.60	177.40	63.60	<0.0001	****
	HDL	34.15	12.55	38.60	18.70	0.0077	**	38.35	18.52	44.00	22.00	<0.0001	****
	Non-HDL	120.60	38.70	139.90	44.70	0.0019	**	104.80	47.90	121.80	58.00	0.0051	**

**Figure 3:**
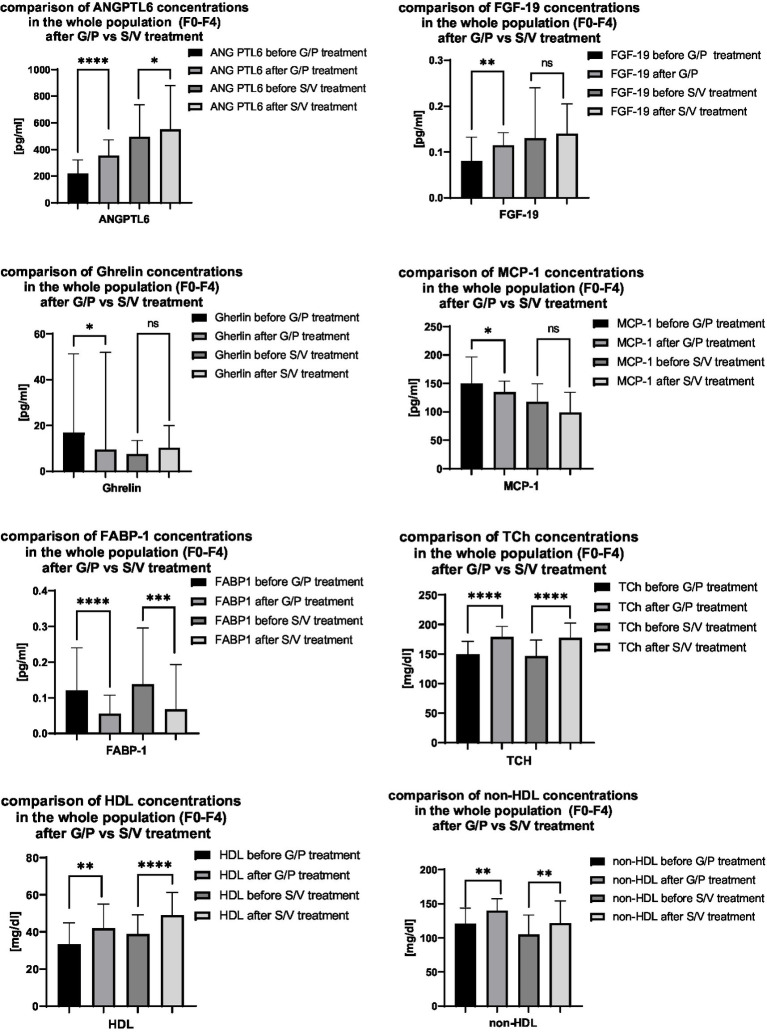
Comparison of selected serum concentrations of ANGPTL-6, FGF-19, ghrelin, MCP-1, FABP-1, total cholesterol, and HDL in the entire patient population (fibrosis stages F0–F4) following treatment with G/P versus S/V. Sample sizes: ANGPTL-6–G/P: *n* = 35, S/V: *n* = 34; FGF-19–G/P: *n* = 34, S/V: *n* = 32; ghrelin–G/P: *n* = 35, S/V: *n* = 35; MCP-1–G/P: *n* = 35, S/V: *n* = 35; FABP-1–G/P: *n* = 35, S/V: *n* = 35; total cholesterol–G/P: *n* = 35, S/V: *n* = 35; HDL–G/P: *n* = 35, S/V: *n* = 34, non-HDL–G/P: *n* = 35, S/V: *n* = 34.

Figure 3 presents a comparative summary of selected serum parameter concentrations before and after treatment with G/P and S/V, irrespective of the degree of hepatic fibrosis. This analysis enables an overall assessment of the biochemical response to both therapeutic regimens across the entire patient cohort, independent of fibrosis stage.

The comparison includes serum concentrations of ANGPTL-6, FGF-19, ghrelin, MCP-1, FABP-1, total cholesterol, HDL, and non-HDL in the entire patient population (fibrosis stages F0–F4) following treatment with G/P versus S/V. Sample sizes for each parameter were as follows: ANGPTL-6–G/P: *n* = 35, S/V: *n* = 34; FGF-19–G/P: *n* = 34, S/V: *n* = 32; ghrelin–G/P: *n* = 35, S/V: *n* = 35; MCP-1–G/P: *n* = 35, S/V: *n* = 35; FABP-1–G/P: *n* = 35, S/V: *n* = 35; total cholesterol–G/P: *n* = 35, S/V: *n* = 35; HDL–G/P: *n* = 35, S/V: *n* = 34; non-HDL–G/P: *n* = 35, S/V: *n* = 34.

3) Concentrations of selected parameters, stratified by individual fibrosis stages (F1, F2, F3, F4), regardless of treatment regimen. The figure presents parameter distributions separately for each stage of liver fibrosis, allowing evaluation of biomarker levels across different degrees of hepatic damage, independent of antiviral therapy used.

**Figure 4:**
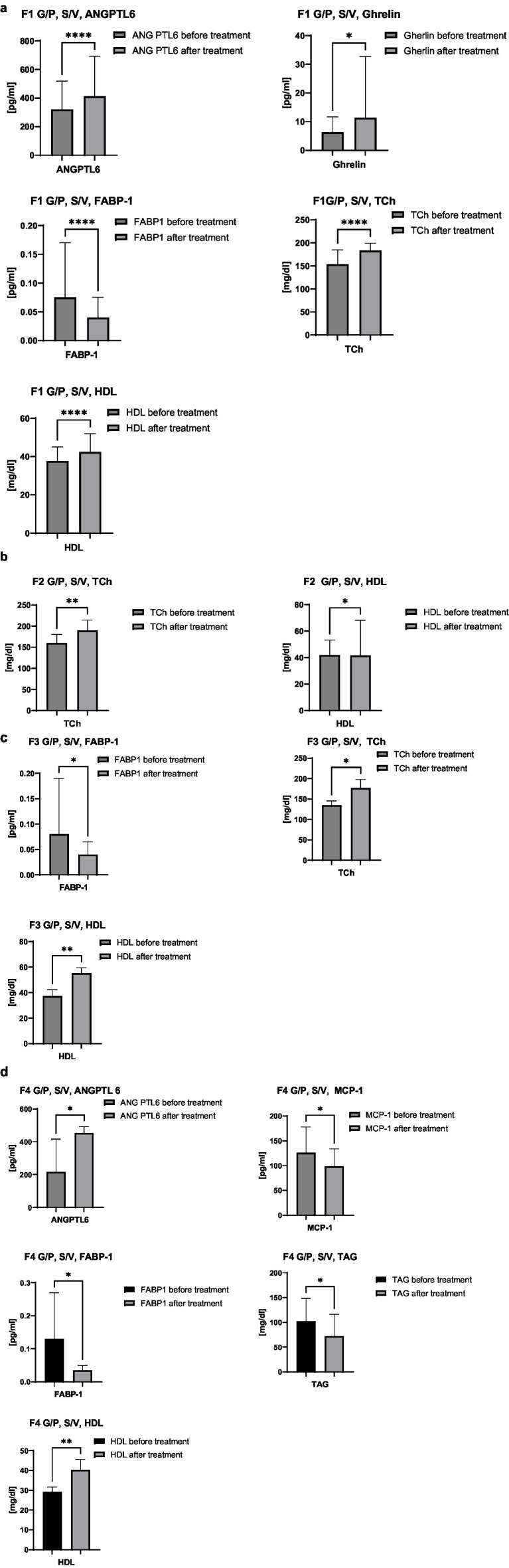
**(a)** Comparison of serum concentrations of ANGPTL-6, ghrelin, FABP-1, total cholesterol, and HDL in patients with liver fibrosis stage F1, regardless of treatment regimen (G/P or S/V). Sample sizes: ANGPTL-6—*n* = 37; ghrelin—*n* = 38; FABP-1—*n* = 38; total cholesterol—*n* = 38; HDL—*n* = 38. **(b)** Comparison of serum concentrations of cholesterol, and HDL in patients with liver fibrosis stage F2, regardless of treatment regimen (G/P or S/V). Sample sizes: total cholesterol—*n* = 8; HDL—*n* = 8. **(c)** Comparison of serum concentrations of FABP-1, cholesterol, and HDL in patients with liver fibrosis stage F3, regardless of treatment regimen (G/P or S/V). Sample sizes: FABP-1—*n* = 9; total cholesterol—*n* = 9; HDL—*n* = 9. **(d)** Comparison of serum concentrations of ANGPTL6, MCP-1, FABP-1, TAG, and HDL in patients with liver fibrosis stage F4, regardless of treatment regimen (G/P or S/V). Sample sizes: ANGPTL6—*n* = 10; MCP-1—*n* = 10; FABP-1—*n* = 10; TAG—*n* = 10; HDL—*n* = 10.

Selected serum parameters were analysed according to liver fibrosis stages F1–F4, irrespective of treatment regimen (G/P or S/V), with F0 patients excluded due to small sample size. This stratification allows evaluation of biomarker changes across fibrosis severity, independent of therapy, as shown in Figures 4a–d. In Figure 4a (F1), serum concentrations of ANGPTL-6 (*n* = 37), ghrelin (*n* = 38), FABP-1 (*n* = 38), total cholesterol (*n* = 38), and HDL (*n* = 38) are presented. Figure 4b (F2) depicts total cholesterol (*n* = 8) and HDL (*n* = 8). Figure 4c (F3) shows FABP-1 (*n* = 9), total cholesterol (*n* = 9), and HDL (*n* = 9). Figure 4d (F4) presents ANGPTL-6 (*n* = 10), MCP-1 (*n* = 10), FABP-1 (*n* = 10), TAG (*n* = 10), and HDL (*n* = 10). This analysis illustrates stage-dependent variations in biomarker concentrations, enabling comparison of biochemical responses across the spectrum of liver fibrosis independent of the applied therapeutic regimen.

4) Concentrations of selected parameters, stratified by individual fibrosis stages (F1–F4) with an additional breakdown by treatment regimen (S/V vs. G/P). The figure presents parameter distributions separately for each fibrosis stage and treatment subgroup (e.g., F1 S/V, F1 G/P, etc.), enabling a more detailed comparison of biomarker levels in relation to both the severity of hepatic fibrosis and the specific antiviral therapy used.

**Figure 5:**
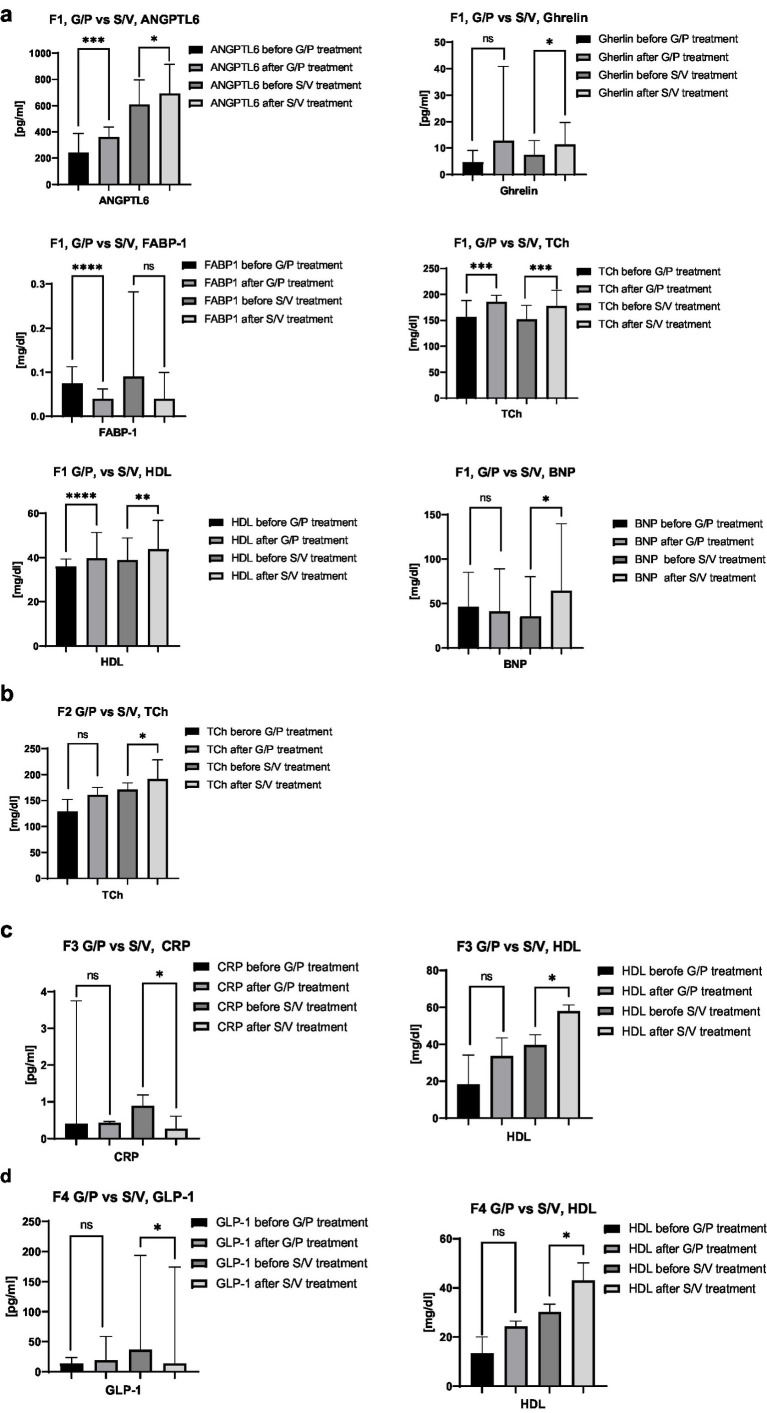
**(a)** Comparison of ANGPTL-6, ghrelin, FABP-1, total cholesterol, and HDL concentrations in patients with liver fibrosis stage F1, stratified by treatment regimen (G/P vs. S/V). The figure illustrates biomarker distribution within each treatment subgroup, enabling evaluation of differences associated with the type of antiviral therapy used. Sample sizes: ANGPTL-6–G/P: *n* = 22, S/V: *n* = 15; ghrelin–G/P: *n* = 22, S/V: *n* = 16; FABP-1–G/P: *n* = 22, S/V: *n* = 16; total cholesterol–G/P: *n* = 22, S/V: *n* = 16; HDL–G/P: *n* = 22, S/V: *n* = 16, BNP– G/P: *n* = 22, S/V: *n* = 16. **(b)** Comparison of total cholesterol concentrations in patients with liver fibrosis stage F2, stratified by treatment regimen (G/P vs. S/V). The figure illustrates cholesterol distribution within each treatment subgroup, enabling evaluation of differences associated with the type of antiviral therapy used. Sample sizes: total cholesterol—G/P: *n* = 4, S/V: *n* = 6. **(c)** Comparison of CRP and HDL concentrations in patients with liver fibrosis stage F3, stratified by treatment regimen (G/P vs. S/V). The figure illustrates biomarker distribution within each treatment subgroup, enabling evaluation of differences associated with the type of antiviral therapy used. Sample sizes: CRP–G/P: *n* = 3, S/V: *n* = 6; HDL–G/P: *n* = 3, S/V: *n* = 6. **(d)** Comparison of GLP-1 and HDL concentrations in patients with liver fibrosis stage F4, stratified by treatment regimen (G/P vs. S/V). The figure illustrates biomarker distribution within each treatment subgroup, enabling evaluation of differences associated with the type of antiviral therapy used. Sample sizes: GLP-1–G/P: *n* = 3, S/V: *n* = 7; total cholesterol—G/P: *n* = 3, S/V: *n* = 7; HDL–G/P: *n* = 3, S/V: *n* = 7.

Serum biomarker levels were analysed according to fibrosis stages F1–F4, with additional stratification by treatment regimen (G/P vs. S/V), allowing detailed comparison of the effects of fibrosis severity and antiviral therapy on biochemical profiles, as presented in Figures 5a–d. In Figure 5a (F1), concentrations of ANGPTL-6 (G/P: *n* = 22, S/V: *n* = 15), ghrelin (G/P: *n* = 22, S/V: *n* = 16), FABP-1 (G/P: *n* = 22, S/V: *n* = 16), total cholesterol (G/P: *n* = 22, S/V: *n* = 16), HDL (G/P: *n* = 22, S/V: *n* = 16), and BNP (G/P: *n* = 22, S/V: *n* = 16) are shown, illustrating biomarker distributions within each treatment subgroup. Figure 5b (F2) depicts total cholesterol (G/P: *n* = 4, S/V: *n* = 6) across treatment groups, enabling evaluation of therapy-related differences. Figure 5c (F3) presents CRP (G/P: *n* = 3, S/V: *n* = 6) and HDL (G/P: *n* = 3, S/V: *n* = 6), highlighting subgroup-specific variations. Figure 5d (F4) shows GLP-1 (G/P: *n* = 3, S/V: *n* = 7), total cholesterol (G/P: *n* = 3, S/V: *n* = 7), and HDL (G/P: *n* = 3, S/V: *n* = 7), demonstrating biomarker distributions by treatment within the most advanced fibrosis stage.

This stratified analysis enables a comprehensive evaluation of biochemical responses in relation to both the severity of hepatic fibrosis and the specific antiviral therapy applied, providing insight into stage- and treatment-dependent differences in biomarker levels.

## Discussion

In the overall study population (fibrosis stages F0–F4), a comparative analysis of serum concentrations of selected biomarkers before and after antiviral treatment with either Glecaprevir/Pibrentasvir (G/P) or Sofosbuvir/Velpatasvir (S/V) revealed several statistically significant changes. An increase in serum levels was observed for ANGPTL-6, FGF-19, ghrelin, total cholesterol, HDL cholesterol, and non-HDL cholesterol indicating potential metabolic or inflammatory shifts in response to antiviral therapy. Conversely, a statistically significant decrease in concentrations was noted for MCP-1 and FABP-1, suggesting a reduction in markers associated with inflammation and fatty acid transport. These findings highlight distinct patterns of biomarker modulation following treatment, potentially reflecting therapeutic effects on liver function and systemic metabolism.

A stratified analysis of serum MCP-1 and FABP-1 concentrations by baseline cholesterol levels (normal vs. elevated >190 mg/dL) revealed key findings. In patients with normal cholesterol (*n* = 64), MCP-1 levels significantly decreased (*p* < 0.05), while no change was observed in those with elevated cholesterol (*n* = 6). FABP-1 decreased in both groups, more prominently in the normal subgroup (***p < 0.0001 vs. *p* < 0.05). These results suggest that lipid status may modulate the anti-inflammatory and metabolic effects of antiviral therapy, with reduced MCP-1 responsiveness reflecting a more resistant inflammatory state. The findings highlight a potential interaction between lipid homeostasis and antiviral response. Additionally, our observations may also suggest that lipid homeostasis may modulate the inflammatory response independently of antiviral therapy, potentially contributing to the development of metabolic-associated fatty liver disease after viral clearance.

Post-antiviral therapy analysis across fibrosis stages (F0–F4) revealed treatment-specific biomarker changes. In the G/P group, ANGPTL-6, FGF-19, total, HDL, and non-HDL cholesterol increased significantly, while ghrelin, MCP-1, and FABP-1 decreased (p < 0.05). The S/V group showed increases in total, HDL, non-HDL cholesterol, and ANGPTL-6, with a decrease only in FABP-1. These findings suggest differing metabolic effects between regimens, with cholesterol and HDL rises indicating improved lipid transport and liver recovery, non-HDL increase pointing to elevated atherogenic lipids, and FABP-1 reduction reflecting better fatty acid metabolism. Decreased ghrelin and MCP-1 after G/P may reflect reduced inflammation, and FGF-19 increase supports enhanced bile acid and lipid regulation. A fibrosis stage-specific analysis was conducted to explore how the severity of liver damage may influence biochemical responses to antiviral treatment, regardless of regimen type (G/P or S/V). Patients with F0 fibrosis were excluded due to limited sample size. The results, presented in Figures 5a–d, demonstrate distinct patterns of biomarker modulation across fibrosis stages F1–F4.

In patients with F1 fibrosis, significant increases in ANGPTL-6, ghrelin, total cholesterol, and HDL were observed, while FABP-1 decreased, indicating early metabolic and lipid transport recovery following viral clearance. In F2 fibrosis, total cholesterol and HDL also increased post-treatment, suggesting an improving lipid profile even in intermediate liver damage, despite the small sample size (*n* = 8). In F3 fibrosis, FABP-1 decreased, and total cholesterol and HDL rose, implying reduced hepatocellular lipid stress and improved metabolism. Among F4 fibrosis patients, ANGPTL-6 and HDL increased, while MCP-1, FABP-1, and triglycerides declined. These changes may reflect vascular remodelling, metabolic normalization, reduced inflammation, and improved systemic lipid balance, even in cirrhosis.

These stage-specific findings underscore the complex, fibrosis-dependent nature of biochemical responses following DAA therapy and emphasize the value of stratified analyses in understanding liver-lipid interactions post-HCV eradication.

A more detailed stratified analysis was conducted to assess how individual fibrosis stages (F1–F4) interact with specific antiviral treatment regimens (G/P vs. S/V) in modulating selected biochemical markers. This approach aimed to disentangle the influence of disease progression from the pharmacologic effects of the antiviral therapies, with the results presented in Figures 5a–d.

In F1 fibrosis, both S/V and G/P therapies improved lipid profiles, but only S/V increased ghrelin and BNP, suggesting broader metabolic effects. In F2 patients, only S/V raised total cholesterol and HDL, implying greater metabolic impact, though small numbers of limit conclusions. In F3, S/V increased HDL and reduced hs-CRP, indicating improved lipid metabolism and inflammation; G/P showed no significant changes. In F4, S/V raised HDL and lowered GLP-1 and triglycerides, suggesting enhanced energy balance, while G/P had no significant effects.

Collectively, this stratified analysis reveals that S/V may elicit broader and more consistent improvements in lipid and inflammatory profiles across fibrosis stages, particularly in more advanced liver disease. The results underscore the importance of tailoring post-therapeutic metabolic monitoring to both fibrosis stage and treatment regimen, especially as some biomarkers such as FABP-1 and HDL appear to reflect differential responses that may be clinically relevant in the context of long-term hepatic and cardiovascular health.

Antiviral therapy with G/P or S/V significantly affects biomarkers of metabolism, inflammation, and fibrosis. S/V induces broader metabolic changes, increasing ANGPTL-6, FGF-19, ghrelin, cholesterol fractions, and reducing FABP-1, GLP-1, triglycerides, and CRP, especially in advanced fibrosis. G/P decreases FABP-1 and MCP-1, reflecting reduced inflammation and liver injury, notably in early fibrosis. Effects were stronger in patients with normal baseline cholesterol, indicating lipid metabolism’s role in treatment response. S/V may better promote hepatic metabolic recovery after HCV clearance, potentially improving long-term outcomes in metabolically at-risk patients. Our findings are supported by previous studies available in the literature and also suggest that successful antiviral therapy for HCV leads to a significant reduction in pro-inflammatory and metabolic biomarkers, including MCP-1 and FABP-1. MCP-1 is a chemokine involved in monocyte recruitment and chronic inflammation, and its decrease may reflect attenuation of hepatic and systemic inflammatory responses following HCV clearance. Similarly, FABP-1, a marker of hepatocellular lipid stress and fatty acid transport, tends to decline after antiviral therapy, likely indicating hepatic regeneration and improved metabolic processing of lipids.

### Potential physiological and pathological mechanisms underlying the observed biomarker changes

The observed increase in ANGPTL6 and FGF-19 may reflect improvement in lipid and energy metabolism following viral eradication. ANGPTL6 has been implicated in enhancing lipid utilization and improving insulin sensitivity, while FGF-19 plays a key role in bile acid signaling, hepatic lipid metabolism, and suppression of *de novo* lipogenesis. Their increase after DAA therapy may therefore indicate a shift toward a more favorable metabolic profile.

The decrease in FABP-1 likely reflects reduced hepatocellular injury and/or hepatic steatosis, as FABP-1 is released into circulation in response to hepatocyte damage and altered intracellular lipid handling. Its reduction after treatment is consistent with improved liver metabolic function following HCV clearance.

Similarly, the reduction in MCP-1 suggests attenuation of systemic and hepatic inflammation. MCP-1 is a key chemokine involved in monocyte recruitment and chronic inflammatory responses; its decrease may indicate reduced inflammatory signaling after elimination of viral-driven immune activation.

Changes observed in ghrelin and GLP-1 further support the concept of metabolic rebalancing after antiviral therapy. Ghrelin is involved in appetite regulation, energy homeostasis, and glucose metabolism, and its modulation may reflect normalization of gut–liver axis signaling following viral clearance. GLP-1 plays a crucial role in insulin secretion, glucose homeostasis, and anti-inflammatory pathways; alterations in GLP-1 levels after treatment may therefore indicate improved incretin response and metabolic control.

Collectively, these biomarker changes suggest that successful DAA therapy may lead not only to viral eradication but also to partial restoration of metabolic, inflammatory, and gut–liver axis homeostasis. These mechanistic considerations have now been incorporated into the Discussion section to better contextualize the biological significance of the observed biomarker changes.

### Potential role of the AMPK/mTOR Axis in biomarker modulation

Although AMPK and mTOR activity were not directly assessed in the present study, several of the analysed biomarkers are known to be functionally linked to cellular energy-sensing and nutrient-signaling pathways involving the AMPK–mTOR axis, which is dysregulated during HCV infection. Experimental studies have demonstrated that HCV suppresses AMPK activity while promoting mTOR signaling, contributing to metabolic and inflammatory alterations in hepatocytes. Biomarkers such as GLP-1 and ghrelin are associated with AMPK-dependent regulation of glucose and lipid metabolism, whereas FGF-19, ANGPTL6, and MCP-1 intersect with PI3K–Akt–mTOR–related metabolic and inflammatory pathways. Therefore, the biomarker changes observed after direct-acting antiviral (DAA) therapy are consistent with a partial restoration of metabolic and inflammatory homeostasis following HCV eradication, potentially reflecting downstream effects of modulation of the AMPK/mTOR axis.

### Cytokines as inflammatory biomarkers in chronic hepatitis C

Cytokines and chemokines act as both prognostic biomarkers and active mediators in viral and metabolic liver diseases. In chronic hepatitis C (HCV), elevated serum levels of MCP-1, IL-6, and IL-10 have been associated with unfavorable treatment outcomes, with MCP-1 reflecting monocyte-derived macrophage recruitment to the liver and correlating with periportal inflammatory activity, while IL-6 is closely linked to liver fibrosis and systemic pro-inflammatory states (22, 39). IL-6 represents a key marker of chronic inflammation, and its biological activity is tightly regulated by a blood-based buffering system in which conventional dendritic cells (cDCs) release soluble IL-6 receptors (sIL-6R), modulating cytokine persistence and trans-signaling during viral challenges (40). Beyond its inflammatory role in HCV, MCP-1 also functions as a metabolically active adipokine, promoting macrophage infiltration, insulin resistance, and hepatic steatosis through increased triglyceride accumulation. Together, these findings highlight the dual immunometabolic role of cytokines as indicators and drivers of disease progression, linking persistent viral infection with chronic inflammation, metabolic dysfunction, and fibrosis (23, 39, 40).

### Natural compounds vs. direct-acting antivirals in HCV therapy

Natural compounds, including silymarin, curcumin, quercetin, glycyrrhizin, and epigallocatechin-3-gallate (EGCG), have demonstrated antiviral activity against HCV by inhibiting viral entry, proteases (e.g., NS3), or RNA replication *in vitro*. They also possess antioxidant, anti-inflammatory, and hepatoprotective effects (41–45). However, clinical efficacy is limited due to low bioavailability, insufficient plasma concentrations, and a lack of large-scale randomized trials (41).

In contrast, direct-acting antivirals (DAAs) such as Glecaprevir/Pibrentasvir (G/P) and Sofosbuvir/Velpatasvir (S/V) target essential viral proteins (NS3/4A, NS5A, NS5B), achieving rapid suppression of viral replication and SVR12 rates >95–99% across all genotypes, including patients with advanced fibrosis or co-morbidities (46–48). DAAs are well-tolerated, with standardized 8–12 week treatment durations, offering clear clinical superiority over natural compounds.

### The reported results may provide a rationale for tailoring antiviral therapy based on the specific metabolic disturbances observed in individual patients

Notably, differences in biomarker responses between G/P and S/V regimens suggest that these treatment strategies may exert distinct metabolic effects. G/P therapy appears to be more strongly associated with reductions in MCP-1 and FABP-1, potentially pointing to a greater anti-inflammatory and hepatoprotective effect in early-stage liver disease. In contrast, S/V treatment is linked to more pronounced improvements in lipid parameters such as HDL and total cholesterol, which may indicate enhanced restoration of lipid homeostasis and reverse cholesterol transport, particularly in advanced fibrosis.

Moreover, baseline lipid status appears to influence the magnitude of biomarker modulation. Patients with normal cholesterol levels at baseline exhibited more substantial decreases in MCP-1 and FABP-1, suggesting that pre-existing dyslipidemia may blunt the anti-inflammatory and metabolic benefits of antiviral therapy. This observation aligns with reports indicating that lipid metabolism and inflammatory signaling are interdependent processes that may influence treatment outcomes in HCV-infected individuals.

These findings underscore the need for further research into the long-term metabolic consequences of antiviral therapy for HCV. While the observed improvements in inflammatory and lipid-related biomarkers are promising, the extent to which these translate into reduced cardiovascular or metabolic risk remains unclear. Stratified post-treatment monitoring based on fibrosis stage and baseline lipid profile may therefore be warranted to optimize long-term outcomes in HCV-cured patients. These findings remain uncertain and require validation in longitudinal outcome-driven studies.

## Study limitations

This study has several limitations that should be acknowledged. First, the observational design and lack of randomization preclude definitive conclusions regarding causal relationships between DAA therapy and the observed changes in metabolic and inflammatory biomarkers. Second, the inclusion of patients with comorbid conditions such as type 2 diabetes mellitus, hypertension, and obesity, although representative of a real-world HCV population, may have independently influenced metabolic and inflammatory parameters, thereby complicating the interpretation of effects attributable specifically to antiviral therapy. Third, the relatively short follow-up period limits the evaluation of the durability of the observed changes and their long-term clinical relevance. In addition, heterogeneity with respect to fibrosis stage, baseline lipid profiles, and antiviral regimens may affect the generalizability of the findings. Finally, the analysis was based on a selected panel of metabolic and inflammatory biomarkers and results interpretation was performed in relation to established reference ranges derived by the manufacturer from healthy populations. Despite these limitations, the study provides valuable insights into the metabolic and inflammatory effects of DAA therapy in patients with chronic HCV infection.

## Data Availability

The original contributions presented in the study are included in the article/supplementary material, further inquiries can be directed to the corresponding author.

## Appendix 1

**Table A1:** Changes in concentration of analysed parameters of G/P -treated and S/V -treated patients regardless of used treatment (*n* = 70).

Parameter [pg/ml]	Median before treatment	Interquartile range before treatment	Median after treatment	Interquartile range after treatment	*p*-value	Statistical significance
ANGPTL6	315.80	336.30	453.30	389.6	<0.0001	****
FGF-19	0.090	0.10	0.13	0.08	0.0344	*
ghrelin	5.070	7.94	7.38	24.29	0.0264	*
MCP-1	133.10	83.78	125.10	57.68	0.0782	ns
FABP1	0.08	0.15	0.04	0.042	0.0005	***

**Table A2:** Changes in parameters concentration of G/P-treated and Sofosbuvir/Velpatasvir-treated patients.

Studied parameter	Median before treatment	Interquartile range before treatment	Median after treatment	Interquartile range after treatment	*p*-value	Statistical significance
TCh (mg/dl)	147.6	50.7	177.5	45.1	<0.0001	****
HDL (mg/dl)	35.55	12.45	43.3	18.92	<0.0001	****
Non-HDL (mg/dl)	115.8	47.6	129.40	43.7	<0.0001	****
CRP	0.31	0.90	0.43	0.69	0.3058	ns
BNP	41.00	63.07	45.23	64.15	0.9022	ns
